# A randomized clinical trial comparing hydrocolloid, phenytoin and simple dressings for the treatment of pressure ulcers [ISRCTN33429693]

**DOI:** 10.1186/1471-5945-4-18

**Published:** 2004-12-15

**Authors:** Mohammad Taghi Hollisaz, Hossein Khedmat, Fatemeh Yari

**Affiliations:** 1 Department of Rehabilitation, Baqyiatollah Hospital, Baqyiatollah University of Medical Sciences, Tehran, Iran; 2 Department of Internal Medicine, Baqyiatollah Hospital, Baqyiatollah University of Medical Sciences, Tehran, Iran; 3 Department of Biostatistics, Lorestan University of Medical Sciences, Khoramabad, Iran

## Abstract

**Background:**

Pressure sores are important and common complications of spinal cord injury. Many preventive and therapeutic approaches have been tried and new trials are evolving. One relatively recent method is application of a hydrocolloid dressing (HD). In this study we compared the therapeutic effects of HD on pressure ulcer healing with two other topical applications, phenytoin cream (PC) and simple dressing (SD).

**Methods:**

Ninety-one stage I and stage II pressure ulcers of 83 paraplegic male victims of the Iran-Iraq war were randomly allocated to three treatment groups. Mean age and weight of the participants were 36.64 ± 6.04 years and 61.12 ± 5.08 kg, respectively. All the patients were managed in long term care units or in their homes for 8 weeks by a team of general practitioners and nurses, and the ulcer status was recorded as "Complete healing", "Partial healing", "Without improvement" and "Worsening".

**Results:**

Complete healing of ulcers, regardless of location and stage, was better in the HD group than the PC [23/31(74.19%) vs 12/30(40%); difference: 34.19%, 95% CI = 10.85–57.52, (P < 0.01)] or the SD [23/31(74.19%) vs 8/30(26.66%); difference: 47.53%, 95% CI = 25.45–69.61, (P < 0.005)] groups. Complete healing of stage I ulcers in the HD group [11/13(85%)] was better than in the SD [5/11(45%); difference: 40%, 95% CI = 4.7–75.22, (P < 0.05)] or PC [2/9 (22%); difference: 63%, 95% CI = 29.69–96.3, (P < 0.005)] groups. Complete healing of stage II ulcer in the HD group [12/18 (67%)] was better than in the SD group [3/19(16%); difference: 51%, 95% CI = 23.73–78.26, (P < 0.005)], but not significantly different from the PC group [10/21 (48%); difference: 19%, 95% CI = -11.47–49.47, (P > 0.05)]. We performed a second analysis considering only one ulcer per patient (i.e. 83 ulcers in 83 patients). This "per patient" analysis showed that complete ulcer healing in the HD group was better than in the PC [20/28(71.4%) vs 11/28 (39.3%); difference: 32.1%, 95% CI = 7.4–56.7, (P < 0.01)] or SD [20/28(71.4%) vs 8/27 (29.6%); difference: 41.8%, 95% CI = 17.7–65.8, (P < 0.005)] groups.

**Conclusion:**

We deduced that HD is the most effective method investigated for treating stage I and II pressure ulcers in young paraplegic men.

## Background

Skin ulcers caused by pressure and strains are known by various names: decubitus ulcer, bedsore, ischemic ulcer and pressure ulcer. "Pressure ulcer", which indicates the etiology of the lesion, seems the most appropriate name [1]. An estimated 50–80% of individuals suffering from spinal cord injury develop pressure ulcers at least once in their lifetime. Most of these ulcers occur during the first two years after injury, but even after 3–4 years an incidence of 30% has been reported [2-4].

Although the major challenge is to prevent the occurrence of ulcers [5,6], therapeutic measures merit due attention. Pressure ulcer therapy is among the expensive of medical and surgical interventions [5-7]. In one study in the United Kingdom, data relating to chronic wound management practice obtained from 15 pressure sore studies showed a cost range of 422–2548 pounds per healed wound for primary dressing, nursing time, wound cleansing and debridements [8]. These figures do not include the much higher costs of hospitalization and plastic surgery. We have tried to find a more effective and cost-efficient method of treatment.

Different methods have been used for preventing and treating pressure ulcers. These include various training programs for patients [4,9,10]; physiotherapy methods employing ultrasound, ultraviolet irradiation and laser treatment [7]; good nutrition emphasizing high protein, high calorie diet and more liquid; electrical stimulation; and application of local ointments and creams such as bacitracin, silver sulfadiazine, neomycin, polymixin, phenytoin and hydrocolloid dressings [11-19].

The results of the studies conducted so far are incompatible, even contradictory. Most of them considered too few patients and/or lacked a control group. In Iran, 5000 patients suffer from spinal cord injury (SCI): of these, 2000 are lran-lraq war victims and 3000 were handicapped by other causes. In view of the enormous prevalence of pressure ulcers in war victims and other spinal handicap patients, and the importance of these lesions in terms of morbidity, mortality and cost of treatment, we have compared the efficacies of applying hydrocolloid dressing, phenytoin cream and a simple dressing. The aims were to determine: 1. which is the most effective in terms of complete ulcer healing; 2. whether healing rates differ with respect to the ulcer stage (I and II) or location (gluteal, ischial, sacral) using these three different methods.

## Methods

The study was a randomized single blind clinical trial involving 2015 Iranian spinal cord injury (SCI) victims of the Iran-Iraq war (1980–1988). The SCI victims were accessed through the mediation and assistance of the Jaonbazan Medical and Engineering Research Center (JMERC) , the medical and research section of the official governmental body responsible for SCI war victims. The study proposal was reviewed, approved and granted by JMERC.

**Figure 1:**
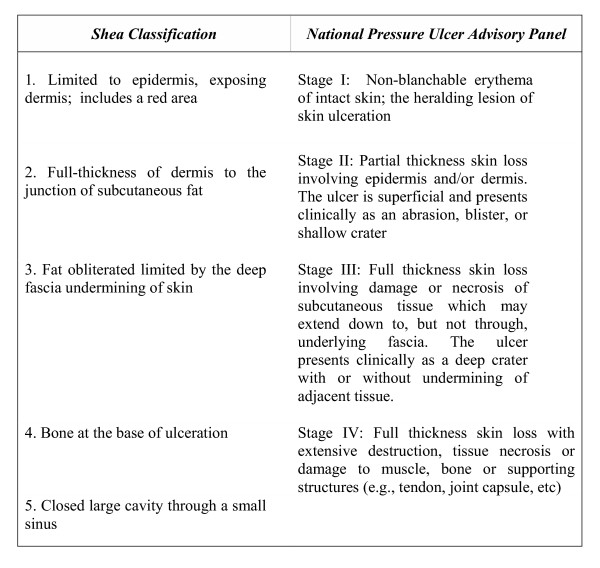
Two pressure ulcer classifications.

The medical records of all 2015 subjects were studied to identify cases with pressure ulcers. Where the data were unknown or unreliable, the patients were visited at home or in victims' long term care centers. Finally, 165 pressure ulcers in 151 patients were identified. All relevant data including patient age and weight, the longevity of the ulcer before our intervention, and the size, stage and location of the ulcer, were collected by the general practitioners in the team. Next, all the patients were examined by one of the authors to confirm their eligibility for the study. The eligibility criteria were: A) Inclusion Criteria: 1. Paraplegia caused by spinal cord injury; 2. Pressure ulcer stage I and II according to Shea classification [20] or National Pressure Ulcer Advisory Panel [21] (Fig. 1); 3. Patient's informed consent; 4. Smoothness of ulcer area to establish whether adhesive could be used at the site. Exclusion criteria: 1. Addiction; 2. Heavy smoking (more than 20 cigarettes a day or more than 10 packs per year; 3. Concomitant chronic disease (e.g. diabetes mellitus or frank vascular disease such as Buerger's disease).

**Figure 2:**
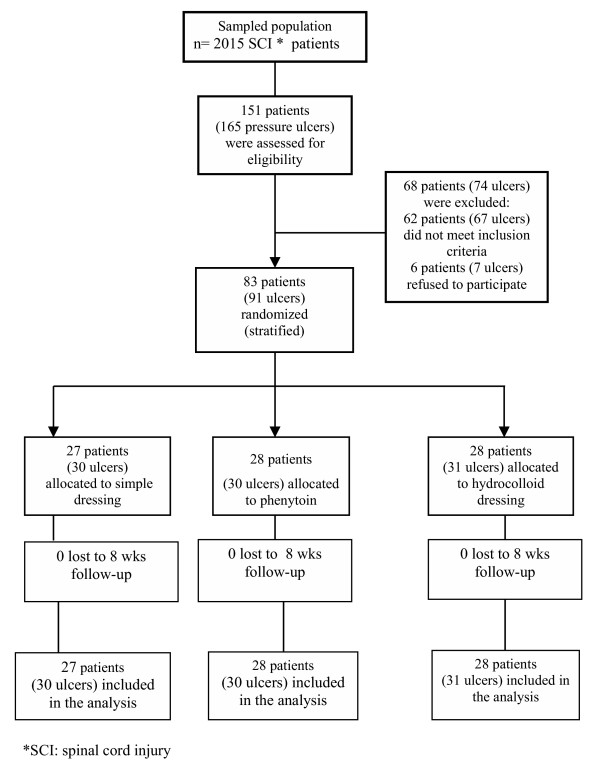
Flow diagram of participants through each stage of the study.

Seventy-four ulcers in 68 patients were excluded because they did not meet these eligibility criteria: 31 ulcers (28 patients) were stage III or higher; 27 ulcers (25 patients) were excluded because of patient's smoking/addiction; 5 ulcers (5 patients) had uneven surfaces; 4 ulcers (4 patients) were excluded because of systemic diseases; and 6 patients with 7 ulcers refused to participate (Fig. 2). Thus, the study sample comprised 83 patients with 91 pressure ulcers in the ischial, sacral or gluteal areas. These 91 ulcers were allocated to three different groups (30 ulcers each) by stratified randomization. Three therapeutic methods were applied as follow: simple dressing (SD), hydrocolloid dressing (HD), and adhesive and phenytoin cream (PC). Two general practitioners and nine nurses trained in treatment interventions administered the protocols.

The SD patients were visited twice a day, the PC patients once a day and the HD patients twice a week. All participants were visited and examined in their family homes or nursing homes by general practitioners every two weeks to ensure that the treatments were being properly applied and were consistent among the three groups. There were no differences in the facilities available for patients in family homes versus nursing homes, and all the patients had free access to victims' long term care centers.

In the SD group, the following steps were taken twice a day. The ulcer was cleaned and washed 3 times with normal saline, then dried with a sterile gauze and, depending on the size of ulcer, covered with wet saline gauze dressing. In the PC group, daily dressing and cleaning of ulcer were similar to the SD group, except that a thin layer of phenytoin cream was applied to the ulcer before the dressing was performed. In the HD group, after the ulcer had been cleaned in a similar manner to the SD group, the hydrocolloid adhesive dressing was applied to the ulcer area. The adhesive dressings were changed twice a week. Any necrotic tissue was debrided before treatment; all debridements preceded ulcer tracing and assignment of the participants to the trial groups. No debridement was allowed after treatment had started. No concomitant topical or systemic antibiotic, glucocorticoid or immunosuppressive agent was allowed during the treatment period. Fortunately, none of our patients needed debridement or the aforementioned concomitant therapies during the study period. There were no differences among the trial groups with respect to other concomitant care measures.

Every two weeks a questionnaire regarding the ulcer's status was completed by the general practitioners; and at the end of 8 weeks, the ulcers' conditions were examined blind by one author and assessed as "Complete Healing", "Partial Healing", "Without Improvement" or "Worsening". To measure each ulcer's surface area, the ulcer borders were traced on to a paper overlay. This primary schematic representation was then scanned, redrawn and measured by AutoCAD 2000 software.

The primary outcome was whether or not the ulcer was completely healed within 8 weeks. "Complete ulcer healing" was defined as: A) For stage I ulcer, intact epidermis, no red area; B) For stage II ulcers, intact dermis and epidermis, no abrasion or ulceration. Other definitions were as follows. "Partial healing" = any decrease in ulcer size compared to the baseline ulcer tracing, excluding complete healing. "Without improvement" = no change in ulcer size compared to the baseline ulcer tracing. "Worsening" = any increase in ulcer size compared to the baseline ulcer tracing. The difference in responses between patients receiving HD and patients receiving the other therapies (PC or SD) were determined [22].

Before the study, we assumed response rates of 30%, 40% and 80% for SD, PC and HD, respectively. Thus, based on the 40% difference, power of 0.85, 95% confidence level and estimated follow-up loss of 10%, 29 patients were required for each study group. The number of ulcers that met the eligibility criteria totaled 91 and all were enrolled in the study.

A random-number table was used to generate the random allocation sequence, and stratified randomization was used to achieve balance between the treatment groups and subgroups (ulcer stages and locations). If a patient had more than one ulcer, all the ulcers were treated by the same method to eliminate the possible complicating factor of treatment interactions. The statistician in the team generated the random allocation sequence. He was informed of the patient list (numbers only) and the ulcer stage and location of each patient.

The treatment category for each patient was determined by the statistician and was delivered in an opaque sealed envelope bearing only the number of the patient. These sealed envelopes were delivered to the general practitioners, along with the list of patients' numbers and names. After each patient was visited, the appropriately numbered envelope was opened by the general practitioner to determine whether the SD, PC or HD method would be used, then the appropriate intervention commenced.

The authors were blind to the patients' assignment to trial groups. The general practitioners were also blind to the treatment of each patient up to the start of the study, when they opened the sealed envelopes. After intervention began, both the general practitioners and the nurses knew the trial groups, because significant differences among the three treatment methods precluded blinding. The patients were also aware of the treatment methods, although they initially had equal chances of entering any of the trial groups. Thus, the study was single-blinded and the author who enrolled the patients to the study was blind to treatment assignment. The author who finally assessed the outcomes was also blind to the trial group of each patient.

To maintain the blinded status on assessment of outcomes, the assessor examined the patients, after the ulcer dressings had been removed by the general practitioners, with no knowledge of the trial groups to which they had been assigned. The gross appearance of the ulcers without dressing, whether healed or not, did not indicate the trial group. The assessor was asked during the 8-week outcome assessment to try to identify which treatment has been administered to each patient. Overall, 27.7% of his guesses were correct (25% in the HD group, 32.1% in the PC group and 25.9% in the SD group), so they were were no better than chance; i.e. there were no significant differences among the three trial groups with respect to proportions guessed correctly (P > 0.2 in all cases).

The study proposal was designed in November 2001, and the recruitment of patients began in March 2002 and lasted about 2 months. Then the patients were allocated to the treatment groups and followed-up for another 2 months. Finally, all the collected data were analyzed within 2 months. Thus the study from proposal to final analysis took about 10 months (November 2001-September 2002). At the end of the study, all the data collected from the patients' preliminary and complementary questionnaires were analyzed by SPSS software using ANOVA and Chi square tests, and P-values of <0.05 were assumed significant. The 95% confidence intervals were also calculated and reported [23]. For rare events (more than 20 percent of cross tabulation cells had values less than 5), Fisher's exact test was used. Based on stage and location of ulcers, subgroup analyses were performed using the same statistical tests.

## Results

**Figure 3:**
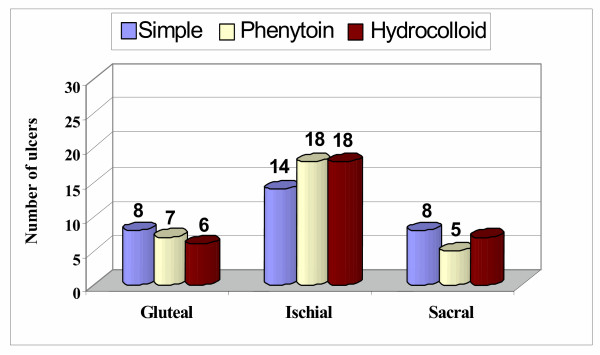
Ulcer distribution according to treatment group and location.

**Figure 4:**
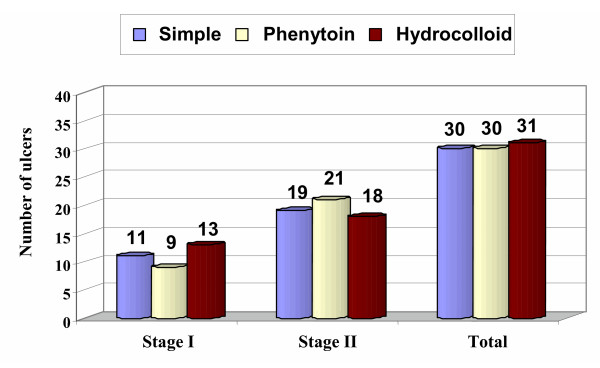
Ulcer distribution according to treatment group and stage.

Ninety-one ulcers in 83 male patients were treated by one of three methods. The mean age and weight of the patients were 36.64 ± 6.04 years and 61.12 ± 5.08 kg, respectively. Of the 91 ulcers, 33 were stage I and the remaining 58 were stage II. There were no significant differences among the three therapeutic groups in baseline demographic characteristics (table 1) or in ulcer location (sacral, gluteal, ischial) or stage (I or II) (Fig. 3 and 4).

**Table 1:** Baseline characteristics of study subjects assigned to hydrocolloid, phenytoin and simple dressing groups

**Variables**	**Mean Age Of patients (yr ± SD)**	**Mean weight Of patients (kg ± SD)**	**Mean duration of ulcer before treatment (wk ± SD)**	**Mean ulcer size (cm^2 ^± SD)**	**Stage of ulcer (no)**	
					
**Treatment group; no**					**I**	**II**
**Total n = 83 patients 91 ulcers**	36.64 ± 6.04	61.12 ± 5.08	6.25 ± 6.56	7.54 ± 12.99	33	58
**Hydrocolloid n = 28 patients 31 ulcers**	36.81 ± 6.71	62.26 ± 5.44	7.63 ± 5.59	7.26 ± 15.4	13	18
**Phenytoin n = 28 patients 30 ulcers**	36.5 ± 4.99	60.07 ± 4.39	5.84 ± 8.04	5.12 ± 3.63	9	21
**Simple dressing n = 27 patients 30 ulcers**	36.6 ± 6.17	61 ± 5.03	5.25 ± 5.39	10.27 ± 15.32	11	19
**P-Value of comparing variables of 3 groups**	P > 0.10	P > 0.10	P > 0.10	P > 0.10	P > 0.62	

The numbers of ulcers and the degree of improvement in the three therapeutic groups are shown in table 2. The completion of healing, regardless of location and stage, was better in the HD than in the PC [23/31(74.19%) vs 12/30(40%); difference 34.19%, 95% CI = 10.85–57.52, (P < 0.01)] or the SD [23/31(74.19%) vs 8/30(26.66%); difference 47.53%, 95% CI = 25.45–69.61, (P < 0.005)] groups. Completion of healing of stage I ulcers in the HD group [11/13(85%)] was also better than in the SD [5/11(45%); difference 40%, 95% CI = 4.7–75.22, (P < 0.05)] or PC [2/9 (22%); difference 63%, 95% CI = 29.69–96.3, (P < 0.005)] groups. Completion of healing of stage II ulcers was better in the HD group [12/18(67%)] than in the SD group [3/19(16%); difference 51%, 95% CI = 23.73–78.26, (P < 0.005)], but there was no significant difference from the PC group [10/21 (48%); difference 19%, 95 CI = -11.47–49.47, (P > 0.05)].

**Table 2:** Healing status of pressure ulcers in 3 treatment groups (hydrocolloid, phenytoin and simple dressing)

**healing status**	**Complete**	**Partial**	**Not improved**	**Worsened**	**Total**
**Treatment group; no (%)**					
**Hydrocolloid n = 31**	23 (74.19%)	4 (12.58%)	2 (6.45%)	2 (6.45%)	31 (100%)
**Phenytoin n = 30**	12 (40%)	4 (13.33%)	12 (40%)	2 (6.66%)	30 (100%)
**Simple dressing n = 30**	8 (26.66%)	5 (16.66%)	8 (26.66%)	9 (30%)	30 (100%)

Gluteal ulcers healed more completely in the HD group [6/6(100%)] than in the PC [2/7 (29%); difference 71%, 95% CI = 37.38–100, (P < 0.005)] or SD [1/8(13%); difference 87%, 95% CI = 63.69–100, (P < 0.001)] groups. The corresponding figures for ischial ulcers were: HD group 13/18(72%) and SD group 3/14 (21%); difference 51%, 95% CI = 21.2–80.7, (P < 0.005)]. The PC group was not significantly different from HD: 8/18(44%); difference 28%, 95% CI = -2.9–58.9, (P < 0.1)]. In the case of sacral ulcers, complete healing in HD group did not differ significantly from either of the others. The results were: HD group 4/7 (57%), SD group 4/8(50%); difference 7%, 95% CI = -50–64.15, (P > 0.35), and PC group 2/5(40%); difference 17%, 95% CI = -39.4–73.4, (P > 0.20)].

We performed a second analysis on 83 ulcers in 83 patients. We selected one ulcer per patient using a random number table; 31 of the 83 ulcers were stage I and the remaining 52 were stage II. There were again no significant differences among the trial groups with respect to baseline characteristics (table 3). This "per patient" analysis showed that complete ulcer healing, regardless of location and stage, in the HD group was better than in the PC [20/28(71.4%, 95% CI = 54.7–88.1) vs 11/28 (39.3%, 95% CI:21.3–57.3); difference 32.1%, 95% CI = 7.4–56.7, (P < 0.01)] or SD [20/28(71.4%, 95% CI = 54.7–88.1) vs 8/27 (29.6%, 95% CI = 12.4–46.8); difference 41.8%, 95% CI = 17.7–65.8, (P < 0.005)] groups.

**Table 3:** Baseline characteristics of study subjects assigned to three trial groups considering the patient as unit of analysis(one ulcer per patient).

**Variables**	**Mean duration of ulcer before treatment (wk ± SD)**	**Mean ulcer size (cm^2 ^± SD)**	**Stage of ulcer (no)**	
			
**Treatment group; no**			**I**	**II**
**Total n = 83**	5.92 ± 6.27	7.78 ± 13.53	31	52
**Hydrocolloid n = 28**	7.12 ± 5.68	7.47 ± 16.4	12	16
**Phenytoin n = 28**	6.11 ± 8.4	5.13 ± 3.67	9	19
**Simple dressing n = 27**	4.47 ± 3.64	10.84 ± 16.32	10	17
**P-Value**	P > 0.20	P > 0.20	P > 0.70	

All completely healed ulcer patients were followed up by monthly visits from general practitioners for a further 4 months after the end of the trial. They were also examined by the assessor author. No recurrence of ulceration was observed in any of the trial groups during this period.

All patients completed the study and there were no losses to follow up, no treatment withdrawals, no trial group changes and no major adverse events (Fig. 2).

## Discussion

Diphenyl hydantoin sodium (phenytoin) is an effective anti-epileptic medication. Its capacity to accelerate ulcer healing was reported more than 40 years ago [24]. Since then, it has been used topically for different kinds of wounds and ulcers such as war wounds, sores caused by venous stasis, atrophic ulcers and burns, and positive effects have been reported [25-27]. Possible mechanisms of action of phenytoin cream on wound healing are as follows: 1. Decrease in serum corticosteroid; 2. Acceleration of assembly and presence of collagen and fibrin in the ulcer area, and stimulation of alkaline phosphatase secretion [28].

The use of HD for healing pressure ulcers dates from about 20 years ago. The benefits of this method in comparison with conventional methods include reduction of bacterial contamination, facilitation of patient movement, improvement in patient's psychological condition, more convenience and less pain [29-34]. Hydrocolloid adhesive dressings absorb water and low molecular weight components from ulcer secretions, so they swell to produce a jelly. This jelly protects the ulcer, and new cells proliferate [35]. Moreover, the jelly stimulates the immune system locally by activating granulocytes, monocytes and the complement system [36], decreasing the effects of bacterial colonization and ensuring autodebridement of the ulcer [1].

Bacterial colonization is likely under the HD layer and is responsible for the unpleasant aroma detected when the dressings are changed, but it should not be misinterpreted as clinical infection. In fact, clinical trials of HD on more than 2000 ulcers have shown a much lower incidence of infection than in other treatment methods [29,30,33]. Thus, the ulcer dry-out method is not considered as useful as it once was, and the current trend is towards a damp method using HD [37-41].

In this study, the therapeutic effects of HD on gluteal and ischial ulcers were shown to be superior to those of PC and SD. In view of the cost of pressure ulcer management in hospitals and sanitariums and the high expense of plastic surgery [42], and the psychological problems associated with paralysis and pressure management in SCI victims [35,43,44], it seems rational to shift to simpler methods that are more cost efficient and executable by the individual patient [31,34,35]. HD treatment of pressure ulcers is less expensive and more comfortable and will ultimately increase the patients' self-confidence [8,45]. These adhesives are available in different sizes and brands convenient for use in ulcers of different parts of body. In the most recent products, the appropriate time for changing the adhesive is indicated by a color conversion. In addition, their transparency makes it easy to observe the ulcer's status without removing the adhesive and dressing [46].

Although the therapeutic effects of HD on sacral ulcers, in contrast to gluteal and ischial ulcers, did not appear in this survey to be significantly better (p > 0.05) than phenytoin and simple dressings, nor was it less effective. Whether the lesser healing effect of HD on sacral ulcers corresponds to the pressure effects in this area, or to greater bacterial colonization or other factors [3,4,6,47], needs to be clarified by further studies.

Gross differences among the three treatment modalities precluded double blinding. Blinding the authors to the treatment groups minimized this limitation. The major tasks, i.e. defining the study population, enrolling the participants who met the eligibility criteria and assessing the primary and secondary outcomes, were performed blind by the authors. To reduce differences in baseline demographic characteristics among the treatment groups and subgroups and to minimize losses to follow-up, war-related SCI patients were recruited and all the patients who met the eligibility criteria were enrolled in the study. They were all relatively young males (mean age 36.64 ± 6.04 years) and had good motivation to complete the course of treatment. The results of this trial cannot be extrapolated to stage III or stage IV pressure ulcers or to other types of wounds. Furthermore, the small numbers of gluteal and sacral ulcers preclude definitive statements about differences among the treatment subgroups.

## Conclusion

The observed efficacy of HD in the treatment of pressure ulcers suggests that it might be effectively applied to other stage I or stage II pressure ulcers.

## Competing interests

The author(s) declare that they have no competing interests.

## Authors contributions

MTH designed the study and wrote the proposal, and visited all the patients and examined them for eligibility criteria. HKH designed the study, helped in the recruitment of patients, planned the data analyses, assessed the trial groups for primary and secondary outcomes and wrote the paper. FY reviewed the literature, advised on data analysis and contributed to writing the paper.

## Funding

The study was supported by the Jaonbazan Medical and Engineering Research Center, the medical and research section of the official governmental body responsible for SCI war victims.

## Pre-publication history

The pre-publication history for this paper can be accessed here:


